# Mobile-Based Augmented Reality Application in Pharmacy Schools Implemented in Pharmaceutical Compounding Laboratories: Students’ Benefits and Reception

**DOI:** 10.3390/pharmacy10040072

**Published:** 2022-06-28

**Authors:** Mohamed Ismail Nounou, Heba A. Eassa, Kamila Orzechowski, Hadeer A. Eassa, Jerry Edouard, Nicole Stepak, Mohammad Khdeer, Mohammed Kalam, Diana Huynh, Eric Kwarteng, Kamilia H. A. Mohamed, Nada A. Helal, Nehal A. Ahmed, Ivan O. Edafiogho, Ola Ghoneim

**Affiliations:** 1 Department of Pharmaceutical Sciences, School of Pharmacy and Physician Assistant Studies, University of St. Joseph, Hartford, CT 06103, USA; nounou@uh.edu (M.I.N.); heassa@usj.edu (H.A.E.); kmorzechowski@usj.edu (K.O.); hadeereassa@outlook.com (H.A.E.); jedouard@usj.edu (J.E.); nstepak@usj.edu (N.S.); mkhdeer@usj.edu (M.K.); mkalam@usj.edu (M.K.); dhuynh@usj.edu (D.H.); iedafiogho@usj.edu (I.O.E.); 2 Eric Kwarteng, PHARMD, Rph, Oncology Pharmacist, Stamford Hospital, Hospital Plaza, Stamford, CT 06902, USA; ekwarteng@stamhealth.org; 3 Faculty of Pharmacy (Girls), Al-Azhar University, Nasr City, Cairo 11657, Egypt; kamilia@azhar.edu.eg; 4 Irma Lerma Rangel College of Pharmacy, Texas A&M Health Science Center, Texas A&M University, 310 Reynolds Medical Sciences Building, Kingsville, TX 77843, USA; nada_helal@tamu.edu; 5 School of Basic Pharmaceutical and Toxicological Sciences, College of Pharmacy, 1800 Bienville Drive, University of Louisiana at Monroe, Monroe, LA 71201, USA; atefkhaledahmedabdn@warhawks.ulm.edu; 6 Department of Pharmaceutical and Administrative Sciences, College of Pharmacy and Health Sciences, Western New England University, Springfield, MA 01119, USA

**Keywords:** pharmacy education, pharmaceutical compounding, augmented reality, student-centered learning

## Abstract

Background: Augmented reality (AR) is a technological approach which combines virtual objects such as text, pictures or videos with physical objects (real-world). The study aimed to design, implement and validate a mobile-based AR application, as a self-paced, interactive, student-centered learning tool be used in the pharmaceutical compounding laboratory course for first year pharmacy students. Method: A mobile-based AR application (Amplified Rx app; HeyPayLess Inc) compatible with iOS and android operating system was developed. A cross-over study design was conducted where alternatively, one group was subjected to ARx app implementation in 8 formulations and the other group served as control. The reception and benefits to students were assessed via a 10 questions survey. In this case, 69 (2019) and 55 (2020) students participated in the study. Result: Students’ use of ARx app was increased in 2020 which indicates its usefulness. For acceptability, leaners enjoyed interactive materials and tutorial videos were the most used and appealing item. Learners described the installation, scanning and operation to be very easy in both years. 86.95% of learners were confident conducting the experiments with the assistance of ARx app in 2019 and increased to 92.73% in 2020. 33.33% considered ARx app to be the most helpful resource in 2019, and the percent was significantly increased to 76.36% in 2020. Conclusion: AR technology implementation in pharmaceutical education could create student-centered engaging and interactive learning experience in fundamental areas such as pharmaceutical compounding laboratories.

## 1. Introduction

Online learning can be defined as learning experiences in structured or unstructured environments using devices (e.g., mobile phones, laptops, etc.) connected to the internet. By which, students can learn and interact with instructors anywhere at any time [1,2]. However, this digitalization faced several logistic and attitudinal challenges. Being dependent on technological devices and internet, those with unreliable internet connections and limited socio-economic levels encounter additional challenges to online learning [3]. Moreover, attitudinal challenges are represented in the lack of face-to-face instructor-student interaction, as well as, classroom socialization which one might argue could affect effective learning [4]. Consequently, educators confronted these challenges, and did their best to become creative with implementing interactive learning approaches using digital platforms [5].

An interactive learning approach is a form of learning and communication between educators and students. It reflects what students know and focuses on students’ needs, abilities, and interests [6]. Based on their knowledge and experience, students can analyze, criticize, gain experiences and new skills. One of the interactive learning methods that represents a student-centered approach is augmented reality (AR).

Augmented reality (AR) is a technological approach which combines virtual objects such as text, pictures or videos with physical objects (real-world) [7]. It may be used to augment traditional teaching modalities such as face to face learning. Its unique ability to create hybrid learning environments in engaging the real world, is the most significant advantage [8]. A recent study by Schneider, et al. 2020 revealed that AR technology allows flexible and repeated access to the content at any time. It has also been suggested that using AR for learning enables students to have a more absorbing and engaging environment without the loss of real world experiences [9].

Akçayır et al., 2017 reported increased implementation of AR in education with 29% directed to higher education [10]. AR has been implemented in different health professions educational sectors such as medicine, dentistry, nursery and pharmacy [9]. Moreover, drug companies and some media resources such as Pharmacy Times have utilized AR applications to provide extra drug information for users. Some studies have attempted to implement AR in pharmacy education such as clinical pharmacotherapy module, learning about specific medication module and sterile compounding training [8,9,11]. However, its application in pharmacy education is still limited [9]. To the best of our knowledge, there is no such application that addresses enhancing learning experience in pharmaceutical compounding labs.

Therefore, the aim of this research project is to develop and implement an augmented reality platform to assess its applicability in pharmaceutical compounding laboratories; PHCY 752) (http://catalog.usj.edu/preview_program.php?catoid=13&poid=2184&hl=%22Pharmacy%22&returnto=search (accessed on 1 June 2022). As a proof-of concept, our objective is to evaluate its ease of use, acceptability and usefulness for student learning. In order to do this, a mobile-based user-friendly application was developed and fed with information derived from PHCY 752 course. Moreover, tutorials videos were created to enhance students’ visualization of the experiments.

## 2. Materials and Methods

### 2.1. Course Description

Pharmaceutical Sciences Laboratory course (PHCY 752) is offered to Pharmacy school first year students at the University of Saint Joseph. The accelerated nature of the program allows this one-credit hour laboratory to be run weekly for 5 consecutive weeks (1 day/week, 6 h/day) during the fall semester. PHCY 752 applies pharmaceutical principles in the formulation of different dosage forms, and develops proficiency in compounding such formulations (solutions, lotions, elixirs, syrups, mouthwashes, suspensions, emulsions, creams, pastes, ointments, gels, troches, lollipops, lip balms, suppositories, capsules and sterile products). Students are to acquire all the aforementioned compounding skills in just 5 weeks (1 day/week).

### 2.2. Development of ARx App

**Figure 1:**
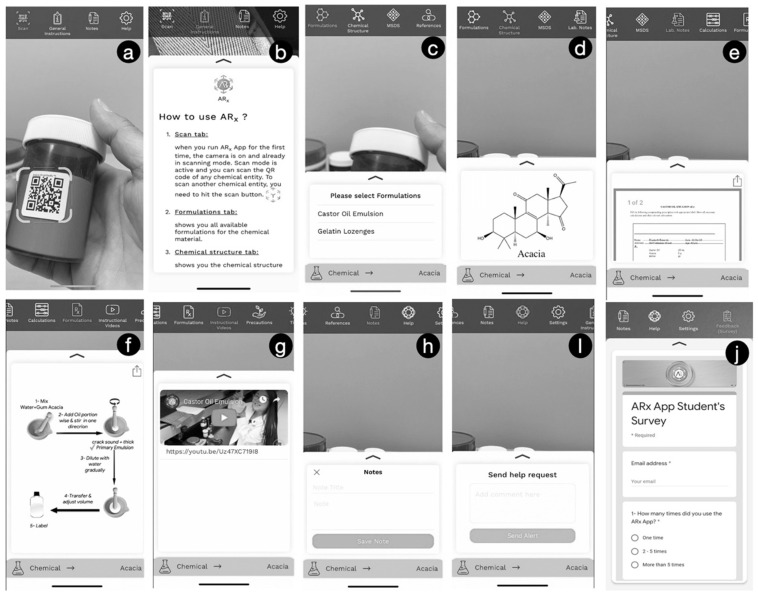
Screenshots of the ARx app demonstrating (**a**) how to scan the QR code, (**b**) general instruction of use, (**c**) formulations available for scanned material, (**d**) chemical structure of scanned material, (**e**) lab notes for selected formulation, (**f**) graphical representation of formulation, (**g**) instructional tutorial video, (**h**) student’s notes, (**i**) help request icon, (**j**) the ARx app student’s survey.

HeyPayLess Inc. “a software company” was contracted to develop the mobile AR application (nonprofit’s purpose). The company developed the core application using swift programming language and Graphical User Interface (GUI) designed to customize and tailor the application to the pharmaceutical laboratory class needs. The application was built to be compatible with both iOS and android operating systems. The iOS version was released on the app store in 2019 followed by an android version on play store in 2020. AmplifiedRx was given ARx symbol to combine augmented reality (AR) with the symbolic pharmacy abbreviation (Rx). The app was fed with database and information that were derived from PHCY 752 course. In this case, 16 compounding formulations and over 70 chemical compounds were included. Data regarding laboratory notes, prescription formulation, calculation, chemical structure (s), graphical representation of the procedures, references, tips, and interactive video tutorials were all included in the database (Figure 1). All data and information about the app are available at http://amplifiedrx.com/ (accessed on 1 June 2022) [12].

### 2.3. Quick Response (QR) Codes Generation

Quick Response (QR) codes were created and placed on each chemical and each piece of laboratory equipment used. These QR codes acted as trigger images and were linked to the ARx app software so that scanning the code would activate all related interactive materials.

### 2.4. Tutorial Videos Creation

Tutorials videos were filmed by a professional crew to provide a visual representation of each formulation. Second year students, who are familiar with the course from their own experience, were selected as actors in these videos under supervision of faculty members. All videos were uploaded to AmplifiedRx YouTube channel [13].

### 2.5. Study Design

The study was designed as a cross over design across students and laboratories. Inclusion criteria included registered first year pharmacy students at the School of Pharmacy, enrolled in the PHCY 752 course. There were no exclusion criteria. Students were divided into two groups and subjected to 16 formulations divided into two clusters (8 formulations/cluster). Each group was subjected to ARx app implementation in only one cluster (8 formulations) where the other group served as control and vice versa. The course was conducted as described per the syllabus. The instructor gave a pre-lab lecture and was present in the lab all the time for any questions/guidance for all students. The study was conducted in two consecutive years; fall 2019 and fall 2020 (which also corresponds to pre COVID and during COVID-19 semesters). In 2020, during COVID-19 semester, the compounding lab was conducted under strict guideline with mask requirements and limited number of students in each laboratory to maintain social distancing. Prior to participation, students were given a detailed explanation about the significance of the study. Students’ consents were collected. Students understood that there was no risk on participating or lack of participating on their grades or academic evaluation. Members of the research team were available in every laboratory to assist in case any technical issues arose.

### 2.6. Outcome Evaluation

By the end of PHCY 752 course, ARx’s usefulness, acceptability and ease of use were evaluated via a survey. Students voluntarily completed a questionnaire that included 4 multiple choice questions, 4 questions of a five-point Likert scale, a 1 yes/no question and 1 open-ended question (Table 1). The study was approved by Institutional Review Board (IRB Protocol Number: 18-0036).

### 2.7. Statistical Analysis

Data for usefulness and acceptability were analyzed statistically via chi-square test and data for ease of use were tested via two-sided fisher’s exact test using GraphPad Prism^®^ version 8.1.1 for MacOS (GraphPad Software, San Diego, CA, USA.

## 3. Results

**Figure 2:**
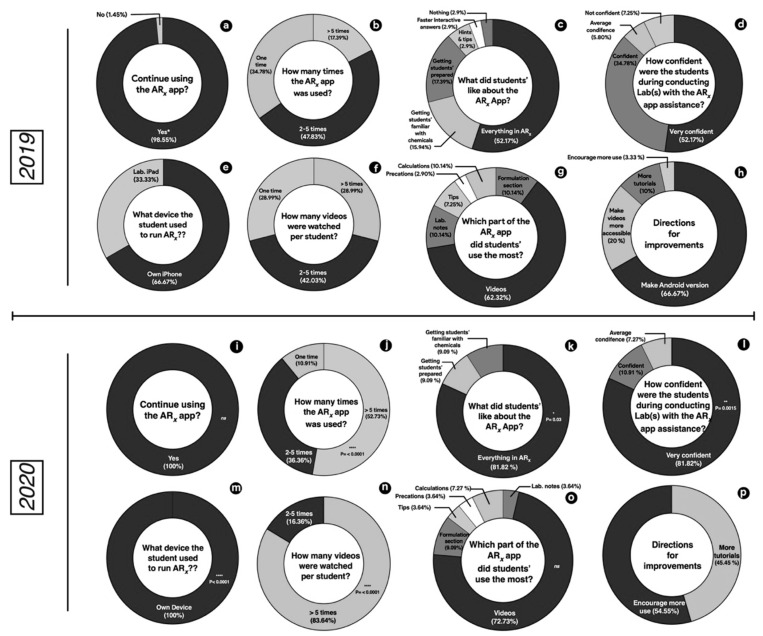
Percentage of learners’ response to survey questions administered to 2019 and 2020 cohorts of PHCY 752 students with respect to continuation of ARx app use (**a**,**i**), times of ARx app use (**b**,**j**), the most enjoyed part of the ARx app (**c**,**k**), degree of students’ confidence during experiment conduction (**d**,**l**), device used to run ARx app (**e**,**m**), number of videos watched per student (**f**,**n**), the most used part of the ARx app (**g**,**o**), and direction for improvement (**h**,**p**). P value indicates the difference significance between data obtained in 2019 and 2020.

In this case, 69 students participated in the fall 2019, while 55 students participated in fall 2020. In 2019, 98.55% of learners agreed that ARx app use in the pharmaceutical compounding labs should be continued (Figure 2a). This percentage has increased (not statistically significant) to 100% in 2020 cohort (Figure 2i). In 2019, learners who used the ARx app >5 times were 17.39% (Figure 2b), whereas 34.78%, and 47.83% of the students used the app for 1 time, and 2–5 times, respectively. Looking at 2020 data, it was observed that the percentages of learners who used the ARx app for one time, and 2–5 times decreased to 10.91%, and 36.36%, respectively, whereas the percentage of learners who opted to use the app >5 times was statistically increased to 52.73% (*p* ≤ 0.001) (Figure 2j).

Overall, 52.17% of the learner appreciated everything provided by ARx app and this percent was significantly increased (*p* ≤ 0.03) to 81.82% of the students in fall 2020 (Figure 2c,k). The majority of learners (86.95%) claimed to be confident conducting the experiment using ARx app (52.17% were very confident, and 34.78% were confident) see Figure 2d. This percentage was sharply increased (significantly higher, *p* ≤ 0.0015) in 2020, where more student learners (92.73%) declared confidence. Of these 81.82% were very confident and 10.91% were confident (Figure 2l). All students (100%) installed the app on their own devices in 2020, compared to 66.67 % in 2019. The remaining 33.33% used the iPads provided in the laboratory (Figure 2e,m).

Videos were the most used item of the app in both years although not statistically significant (62.32% in 2019 and 72.73% in 2020). The calculation section, the tips and precaution sections were also enjoyed by some students (Figure 2g,o). The usefulness of videos was further enhanced in the number of videos watched or self-paced repeated watching (Figure 2f,n). In 2019, 28.99% of the students repeatedly watched more than 5 videos while 42.03% repeated 2–5 videos (Figure 2f). These percentages were significantly (*p* < 0.0001) increased in 2020, with 83.64% of the students repeating more than 5 videos and 16.36% repeating the videos 2–5 times (Figure 2n).

Students were given an open-ended question on their suggestion for improvement. In 2019, large proportion of learners (66.67%) suggested making an android version of the app (Figure 2h), which was taken into consideration and implemented in 2020. Other students suggested making more tutorials (10%) and making videos more accessible (20%). A small number (3.33%) of the students suggested encouragement of use and this suggestion sharply increased to 54.55% in 2020 (Figure 2p).

**Figure 3:**
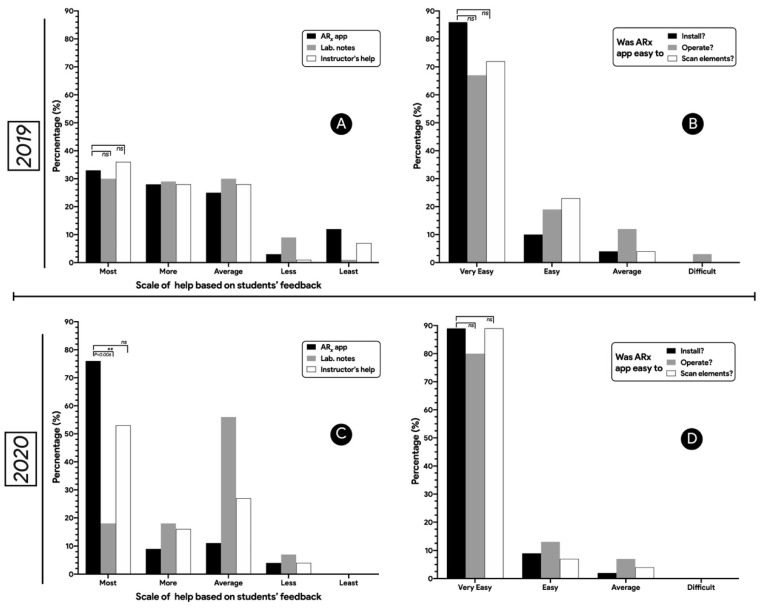
Student response to survey questions administered to 2019 and 2020 cohorts of PHCY 752 students with respect to scale of help (**A**,**C**) and ease of use (**B**,**D**). P value indicates the difference significance between different categories.

Figure 3A,C demonstrated the scale of help based on students’ feedback. It was noticed that 33.33% of the students found ARx app the most helpful resource in 2019 compared to lab notes and instructor’s help and this was increased to 76.36% in 2020. Students rated their satisfaction about ease of installation and scanning QR codes between very easy to average. No student described it as difficult (Figure 3B,D). The ease of installation was rated very easy by a large majority of student learners in both 2019 and 2020 (85.51–89.09%). In addition, scanning QR codes and implementation were considered very easy by 72.46% and 89.09% of the students in 2019 and 2020, respectively. There was no significant difference between students’ ability to scan QR codes, install and operate the ARx app. Table 1 illustrated the actual survey questions and answers in the two cohorts.

## 4. Discussion

As described earlier, the use of AR in pharmacy education is limited, and the use in pharmaceutical compounding laboratories is not a common practice among pharmacy schools [9]. Thus, the interactive Amplified Rx (ARx) app is rather unique. Amplifying learners’ knowledge via a self-paced augmented reality interaction gave the app its name. Although the app was developed in 2019, and despite the fact that the first implementation was in fall 2019, the second implementation in 2020 is what makes this publication unique. The two sets of data we have in hand now (2019 and 2020) could influence the way we use augmented reality in the foreseeable future.

There is no doubt that COVID-19 had its impact on the teaching and learning process worldwide [4,14]. Students and instructors were forced to familiarize themselves with technology due to the instant switch to online learning in almost all schools around the globe. Therefore, more students (and faculty) became more technologically savvy in 2020 compared to 2019 [5]. The authors believe that finding the “go to” lab augmented reality friend in fall 2020, as an additional interactive tool assisted in the engagement process of student learners after students had been on their own for the majority of spring 2020.

Usefulness, acceptability, and ease of installation were the three outcomes measured by the authors in the 10-item survey administered right after implementing the ARx app in the pharmaceutical compounding laboratory course (PHCY 752) to the two cohorts of learners in fall 2019 and fall 2020.

### 4.1. Usefulness

Overall, the vast majorities of 2019 and 2020 learners agreed that the app’s use in the pharmaceutical compounding laboratories should be continued, which is a clear indicator of the app’s usefulness, and its suggested impact on students’ positive learning experience. Similarly, Shinta et al., 2020 stated that implementation of AR technology during the pandemic supported the educational role of SMB II Museum. It succeeded to provide a real experience in cultural study [15].

Another indication of usefulness (especially in COVID-era) could be drawn from the number of times student learners used the app. These numbers significantly increased in 2020 compared to 2019. This indicates that AR could support student-centered learning approach and help students with problem-solving skills [16].

Tutorial videos were very attractive to learners and were the most used item of the app two years in a row, with no statistical difference. Filming the tutorial videos with selected second year pharmacy students was appealing. Learning the required skills from their peers (or from their bigger brothers and sisters) is much more interesting than watching a generic YouTube video. The usefulness of videos was indicated further by the increasing number of videos watched repeatedly per student on their own pace in 2020 compared to 2019 Lastly, a third of students rated the use of the ARx app as a useful resource more than the traditional laboratory notes or instructor’s help in 2019 and this was significantly increased to 76.36% (see Figure 3A,B).

### 4.2. Acceptability

As a measure of acceptability and satisfaction, the majority of student learners in both cohorts appreciated all the perks provided by ARx app including, but not limited to, becoming familiar with the chemicals used, becoming prepared for upcoming experiments, and the faster interactive answers provided by the app which made learning fun. It was similarly reported that 100% of participants found that using AR technology made learning process more engaging, and motivating [8].

The authors were pleased with the significant increase (*p* ≤ 0.03) in student overall acceptability in 2020 compared to 2019 (Figure 2c,k). These findings were supported by a similar study which observed high degree of students’ acceptability of AR produced object in education process [17].

Additionally, students’ acceptability could be attributed again increased use of technology in teaching and learning during the pandemic. It could also be related to the fact that the pandemic compelled both teachers and students to adapt online learning [18]. Consequently in 2020, students were more technologically savvy than their cohorts in 2019 (as described before). Furthermore, please see the limitations section below.

### 4.3. Ease

Majority of students were confident conducting the experiment assisted by the app in both years. Furthermore, all students rated the ease of installation, operation, and scanning QR codes between very easy to average. No student described it as difficult. This Increasing confidence in using the app can be related to both ease and acceptability.

Taken together, our ARx application was capable of providing an innovative, interactive and fun tool to PharmD students, and offering superior educational experience compared to conventional hands-on exposure. Although designed specifically for the pharmaceutical compounding lab, this app can easily be expanded to be used in other disciplines (with advanced preparation and QR code generations) such as patient assessment lab and cosmeceutical lab just to name a few.

## 5. Limitations

The study only evaluated students’ perspective and reception and did not have the ability to evaluate other components within learning-teaching process of pharmaceutical compounding. The relatively small number of students in each cohort, although indicative of the conclusions drawn here, still small enough to make a generalized conclusion of the applicability of the app outside the accelerated program. The authors are in the process of expanding the use of the app to other national and international programs and will report that in the next communication.

The study did not have a baseline of the participants’ technological ability level before participating in the study, nor a comparison baseline between 2019 and 2020 cohorts when it comes to participants’ technological abilities. All students were encouraged to participate regardless of their technological levels, which might have some effect on the results.

## 6. Conclusions

Technology played a radical role in maintaining functional reasonable tools to achieve quality education. Herein, we designed, developed, implemented and validated a novel, innovative, student -centered mobile-based augmented reality application for the use in the pharmaceutical compounding laboratories, where its use is not common. Our app was released by iOS and android. Two cohorts of students in two consecutive years (pre and during COVID) utilized the app and provided their feedback. The app was very well received and learners from both cohorts admired its ease to install and operate. The usefulness, and acceptability became more apparent during COVID pandemic than pre-COVID time. Such AR tools are viable seeds for future immersive educational experiences to enhance the experimental PharmD compounding laboratory.

## Figures and Tables

**Table 1:** Actual survey questions and answers (2019, 2020).

Questions	Answer												Total	
	2019	2020	2019	2020	2019	2020	2019	2020	2019	2020	2019	2020	2019	2020
1—How many times did you use the ARx App?	More than 5 times		2–5 times		One time								69	55
	12	29	33	20	24	6								
2—What device did you use for the ARx App?	Your own iPhone		Lab. provided iPad										69	55
	46	55	23	0										
3—Was the ARx App easy to [Install?]	Very Easy		Easy		Average								69	55
	59	49	7	5	3	1								
3—Was the ARx App easy to [Scan QR codes?]	Very Easy		Easy		Average								69	55
	50	49	16	4	3	2								
3—Was the ARx App easy to [Operate?]	Very Easy		Easy		Average		Difficult						69	55
	46	44	13	7	8	4	2	0						
4—How confident were you during conducting your lab(s) with the ARx App assistance?	5		4		3		2		1				69	55
	36	45	24	6	4	4	4	0	1	0				
5—Which part of the App did you use most?	Lab Notes		Videos		Formulation Section		Tips		Precautions		Calculation Section		69	55
	7	2	43	40	7	5	5	2	2	2	5	4		
6—Did you watch the instructional videos more than once?	More than 5 videos		2–5 videos		1 video		None						69	55
	20	17	29	31	17	7	3	0						
7—During the experiment(s), what was the most helpful resource? [ARx App]	Most		More		Average		Less		Least				69	55
	23	42	19	5	17	6	2	2	8	0				
7—During the experiment(s), what was the most helpful resource? [Lab Notes]	Most		More		Average		Less		Least				69	55
	21	10	20	10	21	31	6	4	1	0				
7—During the experiment(s), what was the most helpful resource? [Asking Instructor]	Most		More		Average		Less		Least				69	55
	25	29	19	9	19	15	1	2	5	0				
8—What did you like best about the ARx App?	All of the above		Made me familiar with the chemicals		Got me prepared for what I was about to do		Gave me hints and tips		Provided faster interactive answers		None of the above		69	55
	38	44	11	0	12	9	4	0	2	2	2	0		
9—Shall we continue using the ARx App in the following years and enhance it?	Yes		No										69	55
	68	55	1	0										
10—How can we improve your experience as a student? (Optional)	Android version		Make the videos more easily accessible		Tutorial		Encourage more use						30	0
	20	0	6	0	3	0	1	0						

## Data Availability

Not applicable.
